# Genome-wide transposon mutagenesis analysis of *Burkholderia pseudomallei* reveals essential genes for *in vitro* and *in vivo* survival

**DOI:** 10.3389/fcimb.2022.1062682

**Published:** 2022-12-23

**Authors:** Yee-Chin Wong, Raeece Naeem, Moataz Abd El Ghany, Chee-Choong Hoh, Arnab Pain, Sheila Nathan

**Affiliations:** ^1^ Department of Biological Sciences and Biotechnology, Faculty of Science and Technology, Universiti Kebangsaan Malaysia, Bangi, Malaysia; ^2^ Bioscience program, Biological and Environmental Sciences and Engineering Division, King Abdullah University of Science and Technology, Jeddah, Saudi Arabia; ^3^ School of Public Health, The University of Sydney, Sydney, NSW, Australia; ^4^ Centre for Infectious Disease and Microbiology, The Westmead Institute for Medical Research, Sydney, NSW, Australia; ^5^ Sydney Institute for Infectious Diseases, The University of Sydney, Sydney, NSW, Australia; ^6^ Codon Genomics, Seri Kembangan, Malaysia

**Keywords:** *Burkholderia pseudomallei*, *Caenorhabditis elegans*, essential genes, TraDIS, *in vivo* survival

## Abstract

**Introduction:**

*Burkholderia pseudomallei*, a soil-dwelling microbe that infects humans and animals is the cause of the fatal disease melioidosis. The molecular mechanisms that underlie *B. pseudomallei’s* versatility to survive within a broad range of environments are still not well defined.

**Methods:**

We used the genome-wide screening tool TraDIS (Transposon Directed Insertion-site Sequencing) to identify *B. pseudomallei* essential genes. Transposon-flanking regions were sequenced and gene essentiality was assessed based on the frequency of transposon insertions within each gene. Transposon mutants were grown in LB and M9 minimal medium to determine conditionally essential genes required for growth under laboratory conditions. The *Caenorhabditis* elegans infection model was used to assess genes associated with in vivo *B. pseudomallei* survival. Transposon mutants were fed to the worms, recovered from worm intestines, and sequenced. Two selected mutants were constructed and evaluated for the bacteria’s ability to survive and proliferate in the nematode intestinal lumen.

**Results:**

Approximately 500,000 transposon-insertion mutants of *B. pseudomallei* strain R15 were generated. A total of 848,811 unique transposon insertion sites were identified in the *B. pseudomallei* R15 genome and 492 genes carrying low insertion frequencies were predicted to be essential. A total of 96 genes specifically required to support growth under nutrient-depleted conditions were identified. Genes most likely to be involved in *B. pseudomallei* survival and adaptation in the *C. elegans* intestinal lumen, were identified. When compared to wild type *B. pseudomallei*, a Tn5 mutant of bpsl2988 exhibited reduced survival in the worm intestine, was attenuated in *C. elegans* killing and showed decreased colonization in the organs of infected mice.

**Discussion:**

The *B. pseudomallei* conditional essential proteins should provide further insights into the bacteria’s niche adaptation, pathogenesis, and virulence.

## 1 Introduction

The minimum set of genes that are required to maintain bacterial viability is known as the essential set. The genes that are required to maintain basic metabolism or growth in the natural environment or host are referred to as essential genes (Tateishi et al., 2020) and loss of these genes is lethal under a particular environmental condition. Studies on gene essentiality can provide insights into the vital cellular functions of a pathogen whilst contributing to the understanding of the mode of bacterial pathogenesis. Meanwhile, a condition-specific essential gene or conditionally essential gene is one that is required only under certain conditions for growth to occur (Chao et al., 2016). Essentiality of these genes is associated with environmental factors and may vary between different conditions. Transposon Directed Insertion-site Sequencing (TraDIS) (Langridge et al., 2009) was introduced to simultaneously assay the role of every gene within a bacterial genome, under particular growth conditions. Using this platform, Goodall et al. (2018) were able to propose the essential genome of *Escherichia coli* K-12. Tn-seq and INSeq are some examples of other Tn-mutagenesis strategies that have been adopted to identify *Burkholderia pseudomallei* (Gutierrez et al., 2015) and *Acinetobacter baumannii* (Wang et al., 2014) virulence factors which are required for *in vivo* survival such as in the lung of a mouse model of infection. Hence, these tools are versatile and can be adopted for both *in vitro* and *in vivo* experimental settings. Use of these high-density screens will enable a broad search for unique virulence determinants and provide insights into the different host-adaptive strategies that pathogenic bacteria have evolved to establish an acute or chronic infection.

The highly pathogenic *Burkholderia pseudomallei* (White, 2003) is a Gram-negative β-proteobacterium ecological saprophytic organism that inhabits soil, rivers, groundwater and rice paddies. Infection by *B. pseudomallei* results in the life-threatening disease, melioidosis. Normally isolated in endemic areas in Southeast Asia and Northern Australia, the presence of the bacteria and the disease are now often reported from other tropical regions in Africa (Steinmetz et al., 2018) and the Americas (Cossaboom et al., 2020). *B. pseudomallei* is a facultative intracellular pathogen and initiates its life cycle by adhering and entering host cells, escapes from the phagosome, completes its replication within the cytosol and finally disseminates to neighbouring cells (Stone et al., 2014). A number of potential virulence factors have been described including cell surface exposed polysaccharides, specialized protein secretion systems, quorum sensing and a lethal toxin (reviewed in Wiersinga et al., 2018; Yip et al., 2020).

*B. pseudomallei*’s complex life cycles, its extensive armoury of virulence factors as well as its versatility in surviving within a broad range of environments has been attributed to its large genome which comprises of two circular replicons (Holden et al., 2004). There is a distinct partitioning of core and accessory functions in these two chromosomes. The coding sequences (CDSs) residing within Chromosome 1 encode proteins that are mainly involved in core functions, such as nucleotide and protein biosynthesis, chemotaxis, and mobility, macromolecule biosynthesis, amino acid metabolism, as well as synthesis of cofactors and transporters. On the other hand, a greater proportion of CDSs encoding accessory functions such as adaptation to atypical conditions, osmotic protection and iron acquisition, secondary metabolism, regulation, and laterally acquired DNA, are found on Chromosome 2.

Soil, groundwater, stagnant creeks, rice paddies and ponds are the natural reservoirs of this free-living environmental bacteria (Lau et al., 2014). Previous studies have also reported on the ability of *B. pseudomallei* to survive in distilled water for up to 16 years without added nutrients (Moore et al., 2008; Pumpuang et al., 2011). This ability implies a bacterial lifestyle where long-term carbon storage in bacterial cells powers a low-rate metabolism (Inglis and Sagripanti, 2006). As water is known as a “common vehicle” for transmitting diseases, prolonged persistence of *B. pseudomallei* in water is a serious threat to public health. However, the mechanisms that permit this infectious bacterium to adapt to the changing state of nutrient availability, have yet to be fully resolved.

Gene essentiality is dependent on the environment that bacteria live in (Peterson and Fraser, 2001). In the event that genes crucial to the bacteria’s adaptation to and survival in a certain environment are disrupted, their growth rates would be reduced or their growth would be completely arrested. Therefore, genetic determinants that are conditionally essential for bacterial growth under a defined environment reveal the lifestyle adopted by bacteria in response to various stresses present in a particular niche. Saturation transposon mutagenesis coupled with next-generation sequencing tools such as TraDIS and Transposon Sequencing have successfully identified essential genes of *B. pseudomallei* strain K96243 (Moule et al., 2014), *B. thailandensis* (Baugh et al., 2013) and *B. cenocepacia* (Wong et al., 2016; Wong et al., 2018). In this study, TraDIS as a genome-wide screening tool was used to identify essential genes that are indispensable for *B. pseudomallei in vitro and in vivo* viability. We determined conditionally essential genes required for *B. pseudomallei in vitro* growth, particularly under nutrient-depleted conditions and extended the study to uncover the *in vivo* fitness determinants associated with *B. pseudomallei* survival and adaptation in an infected host. The genes identified as essential and conditionally essential provide insights into the adaptive strategies exploited by this pathogen to resist adverse host environments whilst establishing an active infection. The use of single-gene mutants validated the importance of these genes as being essential for *in vivo* bacterial survival. Taken together, the identification of the essential proteins will assist in improving our understanding of *B. pseudomallei*’s ability to adapt to different ecological niches and as a resource of potential bacterial targets for antimicrobial drug development.

## 2 Materials and methods

### 2.1 Bacterial and *Caenorhabditis elegans* strains and growth conditions

*B. pseudomallei* strain R15 (Lee et al., 2007) was used to construct the transposon mutant pool. *B. pseudomallei* wild type and mutant strains were routinely grown in Luria Bertani (LB) broth or Brain Heart Infusion (BHI) broth at 37°C with shaking at 250rpm. Tetracycline (100 μg/mL) and gentamycin (4 μg/mL) were used for selection when necessary.

The *C. elegans* strain *rrf3(pk1426);glp-4(bn2)* was propagated and maintained at 16°C on nematode growth medium (NGM) agar plates with *Escherichia coli* OP50 as the standard food source (Wong et al., 2018). The *C. elegans* population was treated with sodium hydroxide and alkaline hypochlorite to release embryos and all for age synchronization of the worms. The nematodes were propagated at 25°C until the young adult stage before they were used for infection with *B. pseudomallei*.

### 2.2 Construction of large-scale transposon mutants

We used the EZ-Tn*5*<TET-1> Insertion Kit (Cat. No. EZ1921T, Epicentre, Illumina) to construct the transposon mutants, Transposome was prepared according to the manufacturer’s instructions and 1 μL of transposome was added to 50 μL of electrocompetent cells followed by incubation on ice for 30 minutes. The cell suspension was transferred to a pre-chilled electroporation cuvette (2-mm-wide gap) (Bio-Rad, Laboratories, California, USA) and pulsed using the Gene Pulser (Bio-Rad Laboratories, California, USA) at 2.5 kV, 25 μF and 200 Ω. Following the electroporation, SOC medium (950 μL) was added to the electroporated cells which were then incubated at 37°C with agitation for 2 hours. The transformed cells were spread on 2X LB agar supplemented with appropriate concentrations of tetracycline and further incubated at 37°C for 24 hours. The number of mutants obtained was estimated by counting the bacterial CFU. Once colonies had been washed with BHI broth and scraped off the plates, they were stored at -80°C in 30% glycerol. Multiple batches of electrotransformations were conducted until 500,000 mutants were obtained. A large-scale transposon mutant library was generated by combining these multiple pools of mutants (input pool). Aliquots of this input pool were stored at -80°C.

### 2.3 Screening of transposon mutants in nutrient-contrasting growth media

Approximately 1x10^10^ mutants from the input pool were inoculated into 50 mL fresh LB broth and grown at 37°C with aeration for 24 hours (T_1_). Subsequently, 50 μL of this culture (T_1_) was inoculated into 50 mL of fresh LB media and grown for a further 24 hours (T_2_) followed by a second passage (T_3_). The mutants were also grown for 24 hours at 37°C in M9 minimal medium (supplemented with 0.4% glucose as carbon source) (T_1_) and then 1 mL of the culture was transferred to fresh medium for another 24 hours (T_2_), followed by a third passage (T_3_) for 24 hours (schematic diagram is shown in **Supplementary Figure S1**). For each output pool, genomic DNA was extracted from 5 mL of bacteria culture.

### 2.4 Infecting *Caenorhabditis elegans* with *Burkholderia pseudomallei* R15 transposon mutants

Approximately 1 x 10^10^ CFU transposon mutants (hereafter, referred to as the input pool) were inoculated into 50 mL LB broth and incubated at 37°C for 16 hours. One hundred microliters of the culture was spread evenly on NGM agar prepared in 6 cm Petri dishes. An additional 5 mL of the culture was reserved as output-LB. The NGM plates spread with the input pool were incubated at 37°C for 24 hours after which they were left to equilibrate to room temperature for a further 24 hours. The bacterial lawn was washed from 1-2 plates and collected as output-NGM. We also considered the possibility of potential changes to the *in vitro* grown population introduced during the bacterial preparation steps which may have led to the loss of some mutants from the input pool that was later subjected to *in vivo* selection. Mutants grown in both LB and NGM were harvested to gauge possible changes to the *in vitro* grown population. To infect *C. elegans* with the mutants, age-matched young adult worms (*rrf3(pk1426);glp-4(bn2)*) were shifted onto the equilibrated input pool mutants that were spread on the NGM agar. Plates were incubated at 25°C over the infection period (**Supplementary Figure S1**).

### 2.5 Harvesting mutants from the *Caenorhabditis elegans* intestine

At 6 and 24 hpi, mutants were harvested from worm intestines (Ooi et al., 2012). At the designated time points, the worms were rinsed with M9 buffer and transferred into microcentrifuge tubes. The tubes were briefly centrifuged at low speed to remove excess buffer. Worms were treated with 25 mM Levamisole (Sigma-Aldrich, USA) to anesthetize them, rinsed three times in 200 μL of 25 mM Levamisole and 1000 μg/mL kanamycin. The worms were incubated for 2 hours to kill the bacterial cells bound to the worm cuticle. Then, worms were washed four times with 200 μL of Levamisole (25 mM) to remove the killed bacteria and residual antibiotics. Worms were homogenized by mechanical disruption using a motorized pestle in 100 μL of 1% Triton X (Sigma-Aldrich, USA). Serial dilutions were performed on the worm lysates and 10 μL of each dilution was spotted on Ashdown agar to estimate the number of intestinal mutants recovered from each tube. The remainder lysate was spread on modified LB agar (3% tryptone, 1% yeast extract and 1.5% bacteriological agar) supplemented with tetracycline and incubated for 24 hours at 37°C. Colonies were soaked with LB broth, scraped off and harvested as *in vivo* output pools (output-6 hpi and output-24 hpi) (**Supplementary Figure 1**).

### 2.6 Preparation of TraDIS sequencing libraries

All input and output pools were used to prepare sequencing libraries using the method previously described (Wong et al., 2016; Wong et al., 2018). In brief, 1μg of genomic DNA was fragmented to 100-1500 bp fragments and the fragments were end repaired and A-tailed with the NEBNext DNA library adapter. The MP_Ad_a and MP_Ad_b oligos were annealed together to prepare the adapter for the A-tailing of the DNA fragments. The adapter-ligated DNA fragments (200 ng) were subjected to amplification over 22 cycles with the Tra_Fp and Tra_Mp_Rp primers which contain unique 7-bp indexes (indicated by N) for sample multiplexing (Meyer and Kircher, 2010). Size selected (200 bp-500 bp) amplified libraries were purified and quantified with the Bioanalyzer and by quantitative real time PCR using primers qPCR_P5 and qPCR_P7. The purified libraries were sequenced on the Illumina HiSeq 2000 platform using the index primer Tra_IndP and the conventional sequencing primer Tra_SeqP. All primer sequences and PCR cycling conditions are available in **Supplementary Table 1**.

### 2.7 Bioinformatics analysis

Sequence reads within the Illumina FASTQ files were filtered based on 10 bases (TAAGAGACAG) matching the 3’ end of the transposon with one mismatch allowed. Matched reads were then edited to remove the transposon tag and the edited reads were mapped to the *B. pseudomallei* R15 reference genome sequence (Ghazali et al., 2021; European Nucleotide Archive: accession number ERS3410623) using SMALT-0.7.2. The exact transposon insertion site was determined using Bio::Tradis (https://github.com/sanger-pathogens/Bio-Tradis). The method of Langridge et al. (2009) was used to assess gene essentiality. Log_2_ likelihood ratios (LLR) were calculated between the essential and non-essential models. If a gene had a LLR of< -2, it was classified as essential; alternatively, if it had a LLR of > 2, the gene was classified as non-essential. The log_2_ fold change ratios of the observed reads were calculated based on Langridge et al. (2009) and used to compare the differences in read abundance between the input pool and *in vivo* output pools. For every gene (g), the log_2_ fold change was calculated as log_2_[(n_g,A_ + 100)/(n_g,B_ + 100)], where n_g,A_ is the number of observed reads in the output pool and n_g,B_ is the number of reads for the input pool (Wong et al., 2016). For genes with very low numbers of observed reads, high scores were smoothed out with a correction of 100 reads. A normal mode was fitted to the log_2_ fold change mode of distribution and according to the fit, *P*-values were calculated for each gene. Genes with log_2_ fold change of< -2 and corrected *P*-value of< 0.05 were considered to be essential for bacteria to grow under a particular condition.

The genes which met the above criteria and proposed as essential genes were subjected to a search against the collection of bacterial essential genes available in the Database of Essential Genes (DEG) (http://origin.tubic.org/deg/public/index.php/; version 14.7, - Oct. 24, 2016) (Luo et al., 2014). The search was performed using BLASTP and the default parameters provided in DEG. From the BLASTP search, similarities at protein-protein level with E-values of 10^−10^ or less were considered matches. Known *B. pseudomallei* virulence factors were retrieved from the Virulence Factors Database (VFDB) (http://www.mgc.ac.cn/VFs/; database version Feb. 11, 2017) (Chen et al., 2005) and the *Burkholderia* Genome Database (http://www.Burkholderia.com) (Winsor et al., 2008).

### 2.8 Construction of selected *Burkholderia pseudomallei* mutants

The deletion mutant of the selected target gene *bpsl3313* was generated using the method described by Wong et al. (2018). The successful deletion of the targeted region was verified with Sanger sequencing. All primer sequences are listed in **Supplementary Table 1**.

For the second selected target gene, *bpsl2988*, the insertion mutant *bpsl2988::Tn5* was obtained from a *B. pseudomallei* strain R15 transposon mutant library (not published) constructed using the EZ-Tn*5*<TET-1> Insertion Kit (Cat. No. EZ1921T, Epicentre, Illumina). The transposon Tn*5* insertion within the *bpsl2988* open reading frame was confirmed and validated *via* Sanger sequencing.

### 2.9 Growth curve analysis

Wild type *B. pseudomallei* R15 and mutant strains were grown in LB broth for 16 hours at 37°C. The cultures were initially adjusted to an OD_595nm_ of 0.5 in LB or M9 minimal medium, after which, 500 μL was transferred into 50 mL fresh media (LB or M9 minimal) and incubated with shaking at 250 rpm at 37°C. Serial dilutions of the cultures were plated every 2 hours to enumerate the number of live bacteria. For each dilution, triplicate aliquots of 10 μL were dropped onto Ashdown agar and plates were incubated for 48 hours at 37°C.

### 2.10 Colony forming unit assay

The CFU assay was performed to determine if the *B. pseudomallei* mutants were able to colonize *C. elegans* compared to the wild type strain. Intestinal bacteria were collected from infected worms using a method described above for harvesting mutants from the infected nematode intestine (Section 2.5). In brief, *B. pseudomallei* wild type and mutant overnight cultures were adjusted to an optical density (OD_595nm_) of ~ 1.5. Twenty μL of each standardized bacterial inoculum was spread evenly on NGM agar in 3.5 cm Petri dishes and incubated at 37°C. The age-matched *rrf3(pk1426);glp-4(bn2)* young adult worms were transferred onto individual wild type or mutant strain lawns on NGM agar. At various time points (6, 24, 48 and 72 hpi), 10 worms were picked from each plate and transferred into microcentrifuge tubes containing 100 μL of 25 mM Levamisole. For each bacterial strain, 3 tubes (technical replicates) with each consisting of 10 worms were prepared. Worms were washed, and a motorized pestle was used to homogenize the worms in 50 μL of 1% Triton X. Serial dilutions were performed on the lysates and each dilution was spotted on Ashdown agar in triplicate. Agar plates were incubated at 37°C and colonies were counted after 48 hours of incubation. The number of bacteria per worm was obtained by dividing the total number of bacterial CFU in 50 μL of worm lysate with the number of worms retained in each tube.

### 2.11 Nematode and mice killing assays

Overnight cultures of the *B. pseudomallei* strains were adjusted to OD_595nm_ = 1.5 and 20 μL of each bacterial suspension was spread evenly on NGM agar in 3.5 cm Petri dishes and incubated as previously described. *E. coli* OP50 was used as a control for the assay. Forty age-matched young adult worms were transferred to the bacterial lawn (triplicate plates for each test strain) and plates were further incubated at 25°C. The number of live and dead worms were scored every 4−6 h. Nematodes were assumed to be dead when they failed to respond to touch (Huber et al., 2004).

Colonies of wild type and mutant strains were inoculated into BHI broth and incubated at 37°C. Overnight cultures were washed twice with PBS, adjusted to an OD_595nm_ of 0.5 (approximately 1 x 10^8^ CFU/mL) and then diluted 1:200 in sterile Phosphate Buffered Saline (PBS). Female BALB/c mice, aged 8 – 10 weeks old, were sourced from the Animal House Facility, Universiti Kebangsaan Malaysia. Mice were maintained under specific-pathogen-free conditions in a positive pressure environment at 20 – 25°C, subjected to a 12-hour light/dark cycle and fed with a protein-enriched diet and water *ad libitum*. Mice were divided into groups of five and for each group, mice were injected intraperitoneally with either wild type or different mutant strains at 1 x10^5^ CFU of bacteria (in 200 μL of PBS). All infected mice were euthanized at 24 hpi by ether inhalation. The lungs, livers and spleens were aseptically removed and individually homogenized in 2 mL of sterile ice cold PBS. Organ homogenates were serially diluted and for each dilution, 10 μL was spotted on Ashdown agar (in triplicate). Colonies grown on plates were counted after 2 days of incubation at 37°C and the bacterial load per organ was determined. The animal experiments were approved by the Universiti Kebangsaan Malaysia Animal Ethics Committee (UKMAEC; FST/2016/SHEILA/23-MAR/732-MAR-2016-OCT-2018) and strictly adhered to the Universiti Kebangsaan Malaysia Animal Ethics Guidelines.

### 2.12 Statistical analysis

The number of colony forming units (CFU) were analysed with GraphPad Prism version 5.04 and the Mann-Whitney U-test was used to determine statistical significance. The doubling time (g) based on growth curves, was calculated from the exponential phase using the formula:


$$\text{g}=\text{t log }2/\left({\text{log N}}_{\text{t}}–{\text{log N}}_{0}\right)$$

where N_0_ = number of CFU at a point during log phase, N_t_ = number of CFU at a different time point during log phase and t = time interval between N_0_ and N_t_. The *in vitro* growth analysis data were expressed as mean ± standard error of the mean (SEM) from at least two independent assays. Statistical analyses were performed using the unpaired, two-tailed Student’s *t*-test. Results obtained from worm killing assays were analyzed using the Kaplan-Meier nonparametric survival analysis in Statview^®^ 5.0.1 (SAS Institute, Inc). All data are presented as mean ± standard deviation (SD) of a representative from at least three independent experiments.

### 2.13 Nucleotide sequence accession numbers

Sequence reads were deposited in the European Nucleotide Archive database with accession number PRJEB13678 and are accessible *via* http://www.ebi.ac.uk/ena/data/view/PRJEB13678. The sample accession numbers are ERS1124824(Input A), ERS1124825 (Input B), ERS1124812 - ERS1124814 (LB_T1_ – LB_T3_), ERS1124818 - ERS1124820 (M9_T1_ – M9_T3_), ERS1124808 (LB), ERS1124809 (NGM), ERS1124810 (6 hpi) and ERS1124811 (24 hpi).

## 3 Results

### 3.1 Construction and sequencing of *Burkholderia pseudomallei* R15 transposon mutant library

A large-scale mutant library was created by electroporating transposome (Tn*5* transposon-transposase complex) into *B. pseudomallei* and this process generated multiple batches of mutants which were pooled to achieve a large-scale library of ~500,000 mutants. This library is henceforth referred to as “input pool” to represent the initial library. A custom sequencing primer was designed to amplify the last 10 bp sequence of the transposon extending into the adjacent bacterial genomic sequence to precisely identify transposon insertion sites (TIS) for each mutant (Langridge et al., 2009). An average of 20 million reads were obtained for each sequencing library, and the 10 bp transposon tag was detected in more than 92% of the total reads. All sequencing reads without the transposon tag were removed before the sequences were mapped. About 78% of the transposon-tagged reads from replicate libraries (Input A and Input B) were uniquely mapped to the *B. pseudomallei* R15 genome. The number of unique TIS identified across the genome was about 413,000 for Input A and 737,000 for Input B (**Table 1**). Correlation analysis revealed a Spearman’s rho correlation coefficient of 0.9855, indicating low variability between the two replicates (Input A and Input B) (**Supplementary Figure S2**). We combined both Input A and Input B reads and this increased the sequencing depth which enhanced the degree of confidence in identifying unique TIS. A total of 848,811 unique TIS were identified which were distributed across the *B. pseudomallei* R15 genome. The highest density of insertions (about 618,812 unique TIS) was detected in chromosome 1 at an average of one TIS every 7 bp. Approximately 229,999 insertions mapped to chromosome 2 at an average insertion gap of 14 bp. This high density of insertions across both chromosomes indicated that the level of saturation within the *B. pseudomallei* genome was sufficient to identify essential genes by a negative selection screen (Seyednasrollah et al., 2015).

**Table 1:** Summary of TraDIS results from *B. pseudomallei* input pools, *in vitro* and *in vivo* output pools.

Output Pools	Total reads	No. of reads with transposon tags (%)	No. of reads mapped to reference genome (%)	No. of unique insertion sites
*B. pseudomallei* Input Pools				
Input A	25,270,175	23,226,661 (91.9)	18,108,433 (78.0)	737,437
Input B	29,547,508	27,383,430 (92.7)	21,739,030 (79.4)	413,073
*in vitro Output Pools*: passages in LB (T_1_ - T_3_)				
LB - T_1_	21,385,246	20,336,592 (95.1)	16,253,781 (79.9)	543,601
LB - T_2_	33,745,217	31,986,480 (94.8)	25,321,622 (79.2)	539,424
LB - T_3_	26,787,873	25,691,733 (95.9)	21,608,771 (84.1)	284,545
*in vitro Output Pools*: passages in minimal M9 (T_1_ – T_3_)				
M9 - T_1_	17,364,583	16,416,659 (94.5)	13,025,407 (79.3)	408,146
M9 - T_2_	24,214,838	23,159,702 (95.6)	19,792,702 (85.5)	350,744
M9 - T_3_	24,786,906	23,844,128 (96.2)	20,794,956 (87.2)	258,038

### 3.2 Identification of the essential genome of *Burkholderia pseudomallei* R15

We anticipated that a lower number or even a lack of transposon insertions (TIS) would be detected in regions that contain essential genes. This is because the disruption of an essential gene would render the insertion mutant bacteria non-viable and thus, most likely to be absent from the constructed mutant library. However, there are essential genes that can still tolerate disruptions at the extreme 3’ and 5’ ends of the open reading frames (Christen et al., 2011; Le Breton et al., 2015). To circumvent the likelihood of these non-disruptive insertions in the 5’ and 3’ ends, we limited our analysis to only TIS located within the sequence spanning the 5%–90% portion of each locus. Next, we normalized the number of TIS identified per gene based on the length of the respective gene to calculate the insertion index of each gene. The insertion indexes were fitted into a bimodal distribution and we obtained two peaks corresponding to essential and non-essential gene sets (**Supplementary Figure S3**). To determine the essentiality cutoff point, we assumed a log2-likelihood ratio of less than -2. Thus, if the insertion index value of a gene is less than the cutoff value, a gene is at least four times more likely to be essential. We avoided any misinterpretation of a gene as “essential” by discarding sequence reads which mapped to more than one region. Using this premise, 79 genes (of which 40% were insertion sequence (IS) and putative transposases) were excluded from the essential list. Based on these strict selection criteria, a list of 492 genes was obtained and these genes are predicted to be essential for *B. pseudomallei* survival on LB agar supplemented with tetracycline (**Supplementary Data Table 1**).

**Figure 1:**
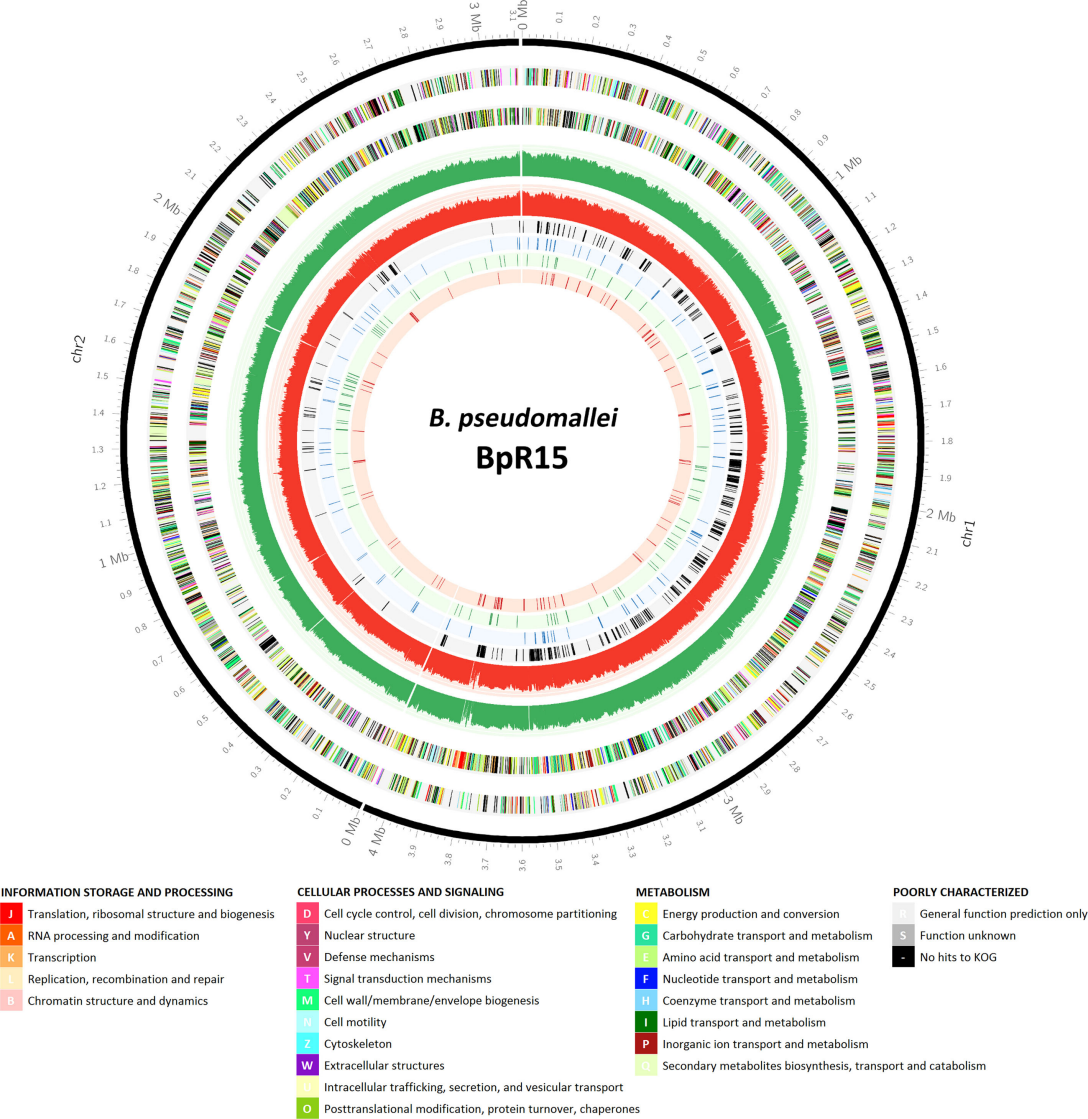
Genomic locations of the candidate essential genes of *B. pseudomallei* R15 and those associated with different *in vitro* conditions. An atlas representation of *B. pseudomallei* R15 genome, rings from outside to inside: track 1, base pair (bp) ruler; tracks 2 and 3, *B. pseudomallei* R15 CDS on the forward and reverse strands, respectively, with colors depicting COG categories (legend shown below); tracks 4 and 5, the number of TIS identified for the input pool over every 1000 bp (green: forward strand; red: reverse strand); tracks 6, 7, 8 and 9, essential genes predicted for input pool, general *in vitro* growth, LB-specific and M9-specific, respectively.

There was a distinct chromosome-level segregation whereby approximately 90% of the 492 genes were located on chromosome 1 (**Figure 1**). This observation is in agreement with the *B. cenocepacia* TraDIS-derived essential data set (Wong et al., 2016) where the *B. cenocepacia* essential genes encoding for core cellular function are also enriched in Chromosome 1. Nonetheless, the lower number of essential genes identified in the *B. pseudomallei* chromosome 2 could be attributed to the distinct essentiality cutoffs applied to each chromosome to offset the imbalanced distribution of TIS. As the average gap between each TIS (14 bp) was larger in Chromosome 2, this meant a potentially higher rate of false discovery in chromosome 2 due to an increased possibility of genes being unrepresented in the transposon library by random chance. The number of genes that can be labelled as “essential” will be reduced with the application of lower essentiality cutoffs (DeJesus et al., 2017).

**Figure 2:**
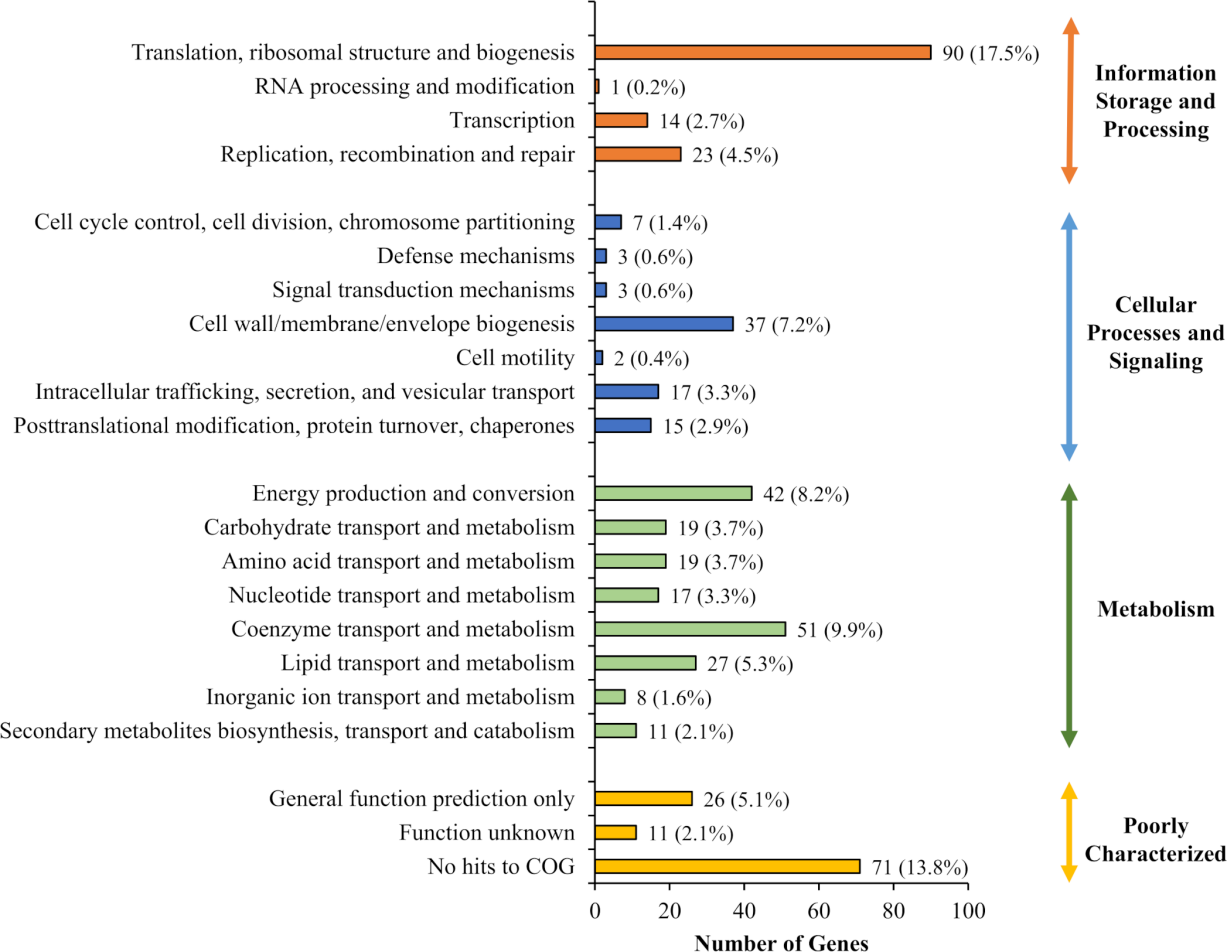
Functional analysis of candidate essential genes of *B. pseudomallei* R15. Distribution of 492 *B. pseudomallei* R15 essential genes (input pool) in each COG functional category.

The identified *B. pseudomallei* R15 essential gene candidates were categorized into their functional classes based on COG (Clusters of Orthologous Groups) designations (**Figure 2**). The distribution of essential genes within each functional category was similar to other different bacteria including *B. cenocepacia* (Luo et al., 2014; Wong et al., 2016) and also the synthetic *Mycoplasma mycoides* JCVI-syn3.0 (Hutchison et al., 2016). Up to 25% of the essential genes encode proteins required for fundamental biological processes necessary to sustain cellular life e.g., enzymes involved in DNA replication, transcription and protein translation. About 7% of the essential candidates fall in the COG functional category “cell wall/membrane/envelope biogenesis”, highlighting the importance of maintaining cell structure and integrity for bacterial viability. In addition, genes involved in metabolism are also important for bacterial survival, with about 10% and 8% of the essential genes in the categories of “coenzyme transport and metabolism” and “energy production and conversion”, respectively. As expected, about 20% of the genes were annotated as encoding for proteins of unknown function or hypothetical proteins.

Among the 492 genes predicted as essential in *B. pseudomallei* R15, 423 genes have homologs in the *B. pseudomallei* reference strain K96243. In a previous TraDIS study, a set of 505 genes were predicted to be essential in *B. pseudomallei* K96243 and these genes and their encoded proteins were proposed as excellent targets for new antimicrobials (Moule et al., 2014). Of these identified K96243 essential genes, 243 were also found to be essential in R15 and this set of genes most likely represents common essential genes shared by *B. pseudomallei* strains. These common core essential genes are enriched in COG categories such as “cell wall/membrane/envelope biogenesis”, “translation, ribosomal structure and biogenesis” and “energy production and conversion” (**Supplementary Table S1**).

**Figure 3:**
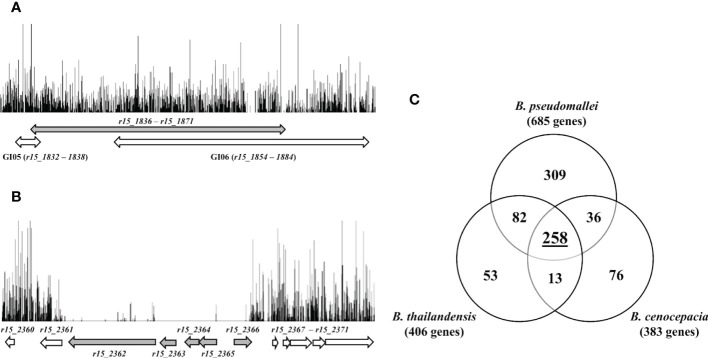
**(A)** Frequency and distribution of transposon insertions in GI05 and GI06 of *B. pseudomallei* R15. Each vertical line represents the position of unique TIS with the height corresponding to the sequencing depth. Grey double-headed arrow indicates homologous region of K96243 genomic island 8 (GI8). White double-headed arrows indicate location of R15 genomic islands (GI05 and GI06). **(B)** Insertions profile of *B. pseudomallei* R15 genomic region *r15_2360 − r15_2371*. Grey arrows indicate essential genes and white arrows indicate non-essential genes. **(C)** Venn diagram shows the overlap numbers of essential genes in three Burkholderia species: *B. pseudomallei*, *B. cenocepacia* and *B. thailandensis*. A total of 258 common Burkholderia core essential genes were derived experimentally using TraDIS and Tn-seq.

Many of the genes located in genomic island 8 (GI8; *bpsl1637* – *bpsl1709*) have previously been predicted as essential in K96243 (Moule et al., 2014). GI8 contains transport proteins, haemolysin-related proteins, amine catabolism proteins, various regulators and YadA-like exported protein (Holden et al., 2004). Interestingly, in R15, homologs of these K96243 genes located within GI8 (*r15_1836* − *r15_1871*) were found to be non-essential. In the R15 genome, these homologous genes are separately located in two genomic islands, GI05 and GI06. As shown in **Figure 3A**, a high density of transposon insertions can be observed in this genomic region (indicated by the grey arrow), implicating that disruption of these GI-associated genes did not impact R15 viability. There is an additional set of R15 essential genes (59) with no homolog in K96243 and hence, is unique to the R15 strain. However, as most of these R15-specific essential genes encode hypothetical proteins, their role(s) in maintaining R15 viability remains to be elucidated. Of particular interest, gene *r15_2362*, which encodes the virulence-associated E family protein VapE, was predicted to be essential, as well as its neighboring genes *r15_2363* (hypothetical protein), *r15_2364* (hypothetical protein), *r15_2365* (hypothetical protein) and *r15_2366* (repressor) (**Figure 3B**). Intriguingly, the VapE gene is also present in other *B. pseudomallei* strains including 1026b, a clinical strain isolated from a septicaemic melioidosis patient blood sample (Johnson et al., 2015). The presence of repressor and terminase encoding genes in this VapE-containing region suggests that this genomic region is likely to be prophage-related. This hypothesis is further supported by the absence of R15_2332 − R15_2376 homologs (~ 40 kb) in K96243, suggesting that this genomic region is not evolutionary conserved but most likely, horizontally-acquired.

### 3.3 Essential genome of the genus *Burkholderia*

*B. thailandensis* (Baugh et al., 2013), *B. pseudomallei* K96243 (Moule et al., 2014) and *B. cenocepacia* (Wong et al., 2016) essential genes were compared to determine the core essential gene set for the *Burkholderia* genus as identified using TraDIS and Tn-Seq. Our TraDIS-derived data set for *B. pseudomallei* R15 was combined with that of K96243 (Moule et al., 2014) into a final repertoire of 685 genes and is hence forth collectively referred to as *B. pseudomallei* essential genes set.

Of the 685 *B. pseudomallei* essential genes, 505 *B. cenocepacia* orthologs were noted but only 294/505 of these *B. cenocepacia* orthologs were previously determined to be essential in *B. cenocepacia* (Wong et al., 2016). When the set of genes was compared with the 406 *B. thailandensis* essential genes (Baugh et al., 2013), we identified 392 *B. pseudomallei* orthologs of which 340 (86%) were deemed essential in the *B. pseudomallei* data set. Taken together, a total of 258 common essential genes were present in *B. pseudomallei*, *B. cenocepacia* and *B. thailandensis*, and most likely represent the core essential genome shared by many species of the *Burkholderia* genus (**Figure 3C**; **Supplementary Table 2**). Not surprisingly, these genes are enriched in the functional categories of “transcription”, “translation, ribosomal structure and biogenesis”, “replication, recombination and repair”, “cell wall/membrane/envelope biogenesis” as well as “energy production and conversion”.

### 3.4 Identification of conditionally essential genes associated with *Burkholderia pseudomallei in vitro* growth

*B. pseudomallei* can thrive in various environmental conditions including nutrient-depleted water (Inglis and Sagripanti, 2006). Identifying genes that are necessary for bacterial growth under a defined condition can reveal the lifestyle adopted by the bacteria. To demarcate the subset of genes which are essential for *B. pseudomallei* growth in nutrient-rich (LB) and nutrient-depleted (M9) media, the input mutant pool was grown in LB and M9 minimal medium over three consecutive passages. At each passage, individual output pools were harvested and subjected to TraDIS analysis to detect any loss of mutants from the initial input pool. This approach allowed mutant selection over continued *in vitro* growth in either the nutrient-rich or nutrient-depleted media. We predicted that mutants of essential genes would be absent in the initial input pool while mutants with reduced fitness (conditionally essential) would be lost only during *in vitro* passages. A summary of TraDIS sequencing results for the *in vitro* grown output pools is shown in **Table 1**.

In this study, we defined genes which are essential for *in vitro* growth as genes for which insertions resulted in fitness defects (i.e., reduced growth rates) and not complete growth arrest as defined for the input pool. Mutants carrying mutations that confer fitness disadvantages would most likely be outcompeted within the mixed population and under-represented after several consecutive passages.

**Figure 4:**
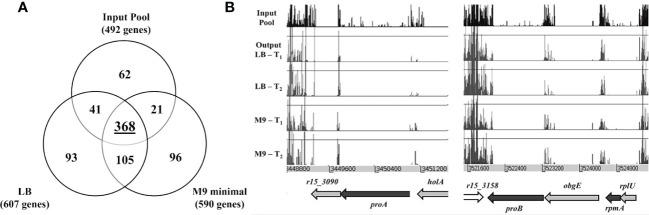
**(A)** Venn diagram presenting the number of *B. pseudomallei* genes classified as essential in (1) initial input pool, (2) nutrient-rich LB media and (3) nutrient-depleted M9 minimal media. **(B)** Insertion profiles of *B. pseudomallei* R15 genomic regions *r15_3090* − *r15_3091* and *r15_3158* − *r15_3162* taken from initial input pool (black) and output pools: LB-T_1_, LB-T_2_ (blue), M9-T_1_ and M9-T_2_ (red). Genes are indicated by arrows, with color coding for gene essentiality: grey, essential genes; black, “general” conditional-essential genes required for *in vitro* growth; white, non-essential gene.

The consensus sets of genes essential for *in vitro* growth was obtained based on genes that were identified as essential in at least two of the three pools of the same media. This approach generated two sets of data corresponding to LB (607 genes) and M9 (590 genes), respectively and this number of genes includes the essential genes predicted for the input pool. We then compared the three different data sets (initial input pool, LB and M9) (**Figure 4A**). There were 368 genes which overlapped and were deemed essential for all conditions and a total of 105 genes were categorized as “general essential genes” critical for bacterial growth *in vitro* as all were conditionally essential under both LB and M9 growth conditions. An additional set of 93 genes is required for *B. pseudomallei* growth in LB but not M9; conversely, 96 genes are conditionally essential when bacteria are grown in nutrient depleted M9 but not LB. These genes were hence classified as “condition-specific” (**Supplementary Data Table 3**). The “general, LB-specific and M9-specific” essential genes locations within the *B. pseudomallei* genome are shown in **Figure 1**.

#### 3.4.1 General *in vitro* growth

Of the 105 “general essential genes” required for *B. pseudomallei in vitro* growth in both LB and M9 minimal medium, about 50% (52/105) has no assigned COG function. Many of the remaining genes (37/105) are involved in the metabolism and transport of carbohydrates, amino acids, nucleotides etc. Disruption of these genes might not cause significant growth arrest but rather, may result in a slower growth rate when the mutant is grown in a mixed population. It is noteworthy that some essential genes may tolerate a small number of insertions and such mutants, whilst unable to grow and divide on the initial selection plate, are still present in the mutant pool at a very low frequency (Langridge et al., 2009). As TraDIS is sufficiently sensitive to detect these insertions, data analysis of the initial mutant pool may inappropriately fail to identify these genes as “essential”. Nonetheless, after the first passage, insertions in these essential genes will be lost. For example, as shown in **Figure 4B**, *proA*, *proB* and *rpmA* failed to be categorized as “essential” in the initial analysis of the input pool, as insertions are still detectable in the coding region of these genes, albeit at a very low frequency. However, following the first passage, mutants with insertions in these essential genes were completely lost from the output pool. Among the conditionally essential genes predicted for “general” *in vitro* growth, 22 genes including *trmE* (tRNA modification GTPase), *ilvC* (ketol-acid reductoisomerase), *ilvD* (Dihydroxy-acid dehydratase), *galU* (UTP—glucose-1-phosphate uridylyltransferase), *proA* (gamma-glutamyl phosphate reductase), *proB* (gamma-glutamyl kinase) and *ahcY* (S-adenosylhomocysteine hydrolase), were previously predicted as essential in the *B. pseudomallei* reference strain K96243 (Moule et al., 2014) strongly suggesting that these genes are indeed part of the *B. pseudomallei* essential genome.

#### 3.4.2 M9-specific

For the M9-specific data set (**Supplementary Data Table 3**), more than 60% of the identified genes (63/96) are involved in metabolic processes, including “amino acid transport and metabolism” (30/63), “coenzyme transport and metabolism” (11/63) and “energy production and conversion” (6/63). The relatively high number of genes associated with amino acid metabolism agrees with that reported for other bacteria grown under nutrient-depleted conditions (Joyce et al., 2006; Lee et al., 2015: Turner et al., 2015). In *P. aeruginosa*, a significant percentage of genes specifically required for growth on minimal MOPS-pyruvate medium are those encoding nucleotide, amino acid and cofactor biosynthetic functions (Lee et al., 2015).

Genes identified were mapped to the KEGG (Kyoto Encyclopedia of Genes and Genomes) pathway database to determine the pathways in which these M9-specific genes and their encoded proteins are involved in. As expected, many of the mapped genes encode proteins required for amino acids biosynthesis. Additionally, genes *cysN*, *purF*, *purH*, *purM* and *purN* which encode proteins involved in purine metabolism were deemed conditionally essential in *B. pseudomallei*, highlighting the importance of *de novo* synthesis of purine nucleotides when purine is limited in a nutrient-depleted environment. The low bioavailability of nucleotide precursors in human serum forces invading pathogens like *E. coli* and *S. enterica* to rely on *de novo* nucleotide biosynthesis, therefore these nucleotide biosynthesis proteins may represent potential targets for antibiotics to treat bloodstream infections (Samant et al., 2008).

Three genes *nadA*, *nadB* and *nadC* involved in the nicotinate-nicotinamide metabolism pathway (KEGG) were also predicted to be conditionally essential for *B. pseudomallei* growth in M9 minimal. Coenzyme NAD (nicotinamide adenine dinucleotide), NADH, NADP, and NADPH, function as hydride acceptors and donors to facilitate various cellular redox reactions (Gazzaniga et al., 2009). NadA (quinolinate synthetase A), NadB (L-aspartate oxidase) and NadC (nicotinate-nucleotide pyrophosphorylase) catalyze the three enzymatic steps that convert amino acid L-aspartate to nicotinic acid mononucleotide, which is then followed by an additional two enzymatic steps to complete the synthesis of NAD. Furthermore, nicotinic acid, which can be sourced from outside the cells, can also be used to produce nicotinic acid mononucleotide. In the absence of nicotinic acid in minimal media, it is reasonable that cells require *nadA*, *nadB* and *nadC* to synthesize NAD *de novo*.

#### 3.4.3 LB-specific

Proteins encoded by 93 genes are required specifically when *B. pseudomallei* is grown in nutrient-rich LB media (**Supplementary Data Table 3**). More than 50% of the encoding genes (55/93) have no assigned COG function, whilst the remainder are enriched in the functional categories of “cell wall/membrane/envelope biogenesis” and “translation, ribosomal structure and biogenesis”. Examples of these LB-specific essential genes are *r15_3385*, *r15_3386* and *r15_3391* (corresponding to *bpsl3179*, *bpsl3180* and *bpsl3184* in K96243). Insertions in these genes affect bacterial growth in LB but not in M9 minimal media. These genes encode proteins vital for post-translational maturation of heme-containing *c*-type cytochromes, which are subunits of electron transport chain components (*bc*_1_ complex and *cbb*_3_ cytochrome *c* oxidase). *Burkholderia* species are thought to employ a type II cytochrome *c* maturation (CCM) system that is well characterized in *Bacillus subtilis* (ResA, ResB, ResC, CcdA) and *Bordetella pertussis* (CcsA, CcsB, CcsX, DipZ) (Beckett et al., 2000; Le Brun et al., 2002). Cytochrome *c* biogenesis proteins encoded by the *r15_3385* (*bpsl3179*) and *r15_3386* (*bpsl3180*) genes are homologous to ResB (also known as CcsA) and ResC (also known as CcsB) respectively. ResB/CcsA binds heme in the cytoplasm and exports it to the extracellular domains of ResC/CcsB, which is responsible for the covalent ligation of heme to apocytochrome *c*. The disulfide bond in the heme-binding motif of apocytochrome *c* must be reduced to allow thioether bonds formation and heme attachment. This process involves the two redox-active proteins, CcdA/DipZ and ResA/CcsX (Travaglini-Allocatelli 2013). DsbD, a transmembrane thioredoxin protein encoded by *r15_3391* (*bpsl3184*), is homologous to CcdA.

### 3.5 Screening of a transposon mutant pool in *Caenorhabditis elegans* identifies genes required for *Burkholderia pseudomallei* survival and adaptation *in vivo*

*B. pseudomallei* wild type transposon mutant pools were used as the input agents to infect *C. elegans*. The input pools were initially cultured in LB medium and then spread on Nematode Growth agar (Tan et al., 1999). As mentioned earlier, the multi-step process to prepare the bacteria could have introduced selection pressure on the mutant population. Hence, there was a possibility that some mutants were already lost from the initial input pool before this pool was subjected to any *in vivo* selection. We assessed for this loss by analyzing mutants grown in LB and NGM which are referred to as output-LB and output-NGM (*in vitro* output pools).

A large-scale infection assay was performed using thousands of worms per experiment. Intestinal mutants were recovered from a population of worms at 6 hrs and 24 hrs post-infection (hpi). The infection experiments were conducted separately for a total of four times, and a mutant pool of approximately 1 x 10^7^ mutants were obtained for each *in vivo* output pool (6 hpi and 24 hpi). TraDIS libraries of *in vitro* and *in vivo* output pools were sequenced with an average of 25 million reads generated for each library of which, > 94% of the total reads contained the correct 10 bp transposon tag. Over 62% of the tagged reads were uniquely mapped to the *B. pseudomallei* R15 genome, resulting in 480,000 - 690,000 unique TIS identified across both chromosomes (**Table 2**). For output-6 hpi and output-24 hpi, one TIS was detected every 14 bp and > 50 reads were available for each insertion. This high-density genome saturation is sufficient to estimate the relative abundance of mutants before (input pool) and after *in vivo* selection (output pool). A gene was important or costly for bacterial fitness in the infected host if the abundance of relative reads was at least four times lower in the *in vivo* output pool (log_2_ fold change< -2; adjusted p-value<0.05) than that in the initial input pool.

**Table 2:** Summary of TraDIS results from *B. pseudomallei in vivo* output pools.

Output Pools	Total reads	No. of reads with transposon tags (%)	No. of reads mapped to reference genome (%)	No. of unique insertion sites
*in vivo Output Pools*: *C. elegans* model (LB, NGM, 6 hpi, 24 hpi)				
LB *(in vitro)*	23,336,769	22,206,977 (95.2)	13,775,244 (62.0)	639,031
NGM *(in vitro)*	25,653,132	24,513,609 (95.6)	21,209,514 (86.5)	689,408
6 hpi *(in vivo)*	34,794,847	33,164,835 (95.3)	25,537,923 (77.0)	482,078
24 hpi *(in vivo)*	37,767,762	35,682,814 (94.5)	25,951,458 (72.7)	504,465

**Figure 5:**
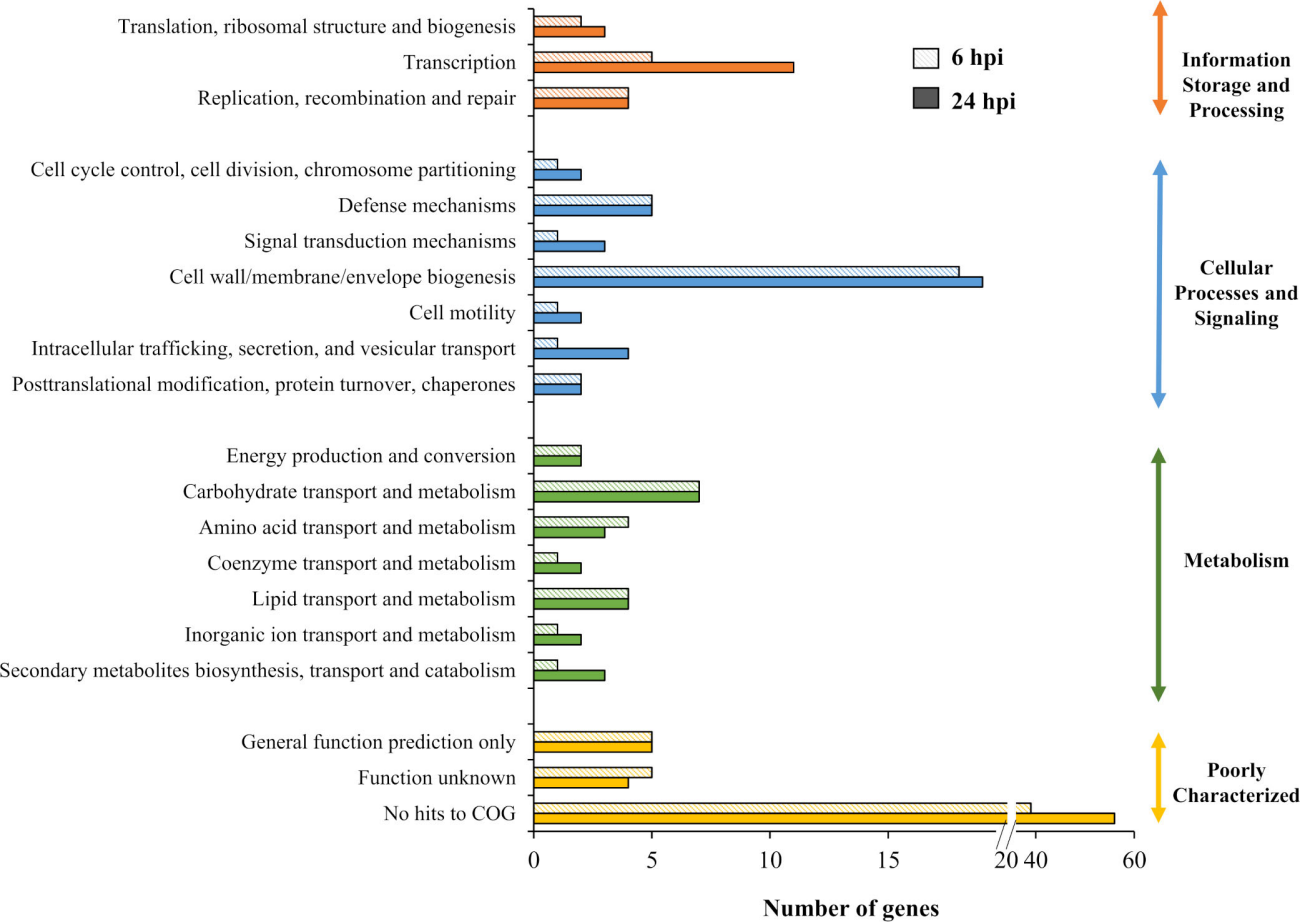
Distribution of genes required for *B. pseudomallei* survival *in vivo* based on COG functional category. The number of genes identified for each category was plotted for *in vivo* output pools collected at 6 and 24 hours post-infection (hpi).

One hundred and thirty genes from output-6 hpi and 180 genes from output-24 hpi met the criterion of log_2_ fold< than -2. The M9-, LB- and NGM-specific conditionally essential genes required for growth *in vitro* were pooled and removed from the list of genes required for bacterial survival *in vivo*. Therefore, 98 genes were required for *B. pseudomallei* survival in the *C. elegans* intestinal lumen during early infection (6 hours post-infection) while 130 genes were deemed important for survival up to 24 hpi. Of the two sets of genes, 84 genes were important for *B. pseudomallei in vivo* fitness throughout the worm infection period (6 and 24 hours post-infection) (**Supplementary Data Table 4**). Genes that negatively impacted *B. pseudomallei* survival *in vivo* were classified based on the COG annotation system. As depicted in **Figure 5**, about 50% of the genes encode poorly characterized proteins with no assigned COG function, hence, how the encoded proteins contribute towards *B. pseudomallei* fitness in the infected host remains unclear. Approximately 30% of the genes were categorised as “cell wall/membrane/envelope biogenesis”, “transcription”, “carbohydrate transport and metabolism” as well as “defence mechanisms”.

### 3.6 Validation of TraDIS results using single-gene knockout mutants

To validate the TraDIS-derived *in vivo* data, mutants of two selected genes (*bpsl2988* and *bpsl3313*) were obtained. These two genes are deemed essential for *in vivo* survival at both 6 hpi and 24 hpi, with the numbers of unique TIS significantly reduced by more than 60-fold when compared with the input pool (**Table 3**). *bpsl3313* encodes a H-NS-like transcriptional regulator while *bpsl2988* encodes a putative sugar kinase. In a previous study, we screened a library of *B. pseudomallei* R15 Tn5 transposon mutants in a *C. elegans* infection model and identified *bpsl2988* as one of the genes with significant attenuation in worm killing as compared to the wild type (data not published). The ability of the single-gene deletion mutants (Δ*bpsl3313*) and Tn*5* insertion mutant (*bpsl2988*::Tn*5*) to survive and proliferate in the *C. elegans* intestine was measured by enumerating the number of *in vivo* colonizing bacteria at 6 hours and 24 hours post-infection.

**Table 3:** *B. pseudomallei* genes selected for validation.

Gene	Product	COG category	Mutant reduction fold change (6 hpi)	Mutant reduction fold change (24 hpi)
*bpsl3313*	HNS-like transcriptional regulator	DNA-binding protein H-NS	65.50	65.50
*bpsl2988*	sugar kinase	Sugar kinases, ribokinase family	156.28	121.14

**Figure 6:**
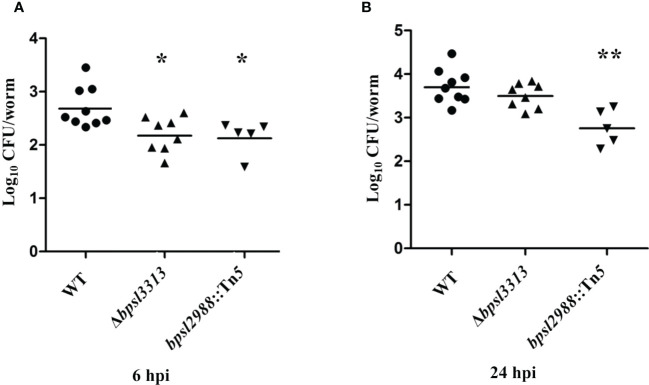
Colonization ability of *B. pseudomallei* wild type (WT) and mutants in *C elegans* intestinal lumen at **(A)** 6 hours and **(B)** 24 hours post-infection (hpi). Each marker corresponds to the average bacterial CFU (log_10_) extracted from 10 infected worms with horizontal lines signifying the geometric mean. The asterisks represent *p* values of the WT counts versus the mutants (*t*-test, *p<0.05, **p<0.01).

As shown in **Figure 6**, during early infection (6 hpi), intestinal bacterial loads in worms infected with Δ*bpsl3313* and *bpsl2988*::Tn*5* mutants were significantly lower (*t*-test, *p*<0.05) as compared to the wild type (WT) strain. After prolonged infection (24 hours), only *bpsl2988*::Tn*5* showed limited intestinal colonization (*t*-test, *p*<0.01), whilst the ability of Δ*bpsl3313* to survive in the worm intestine was comparable to that of the wild type (**Figure 6**). Insertions in these two genes negatively impacted mutant fitness in the worm intestine at both 6 hpi and 24 hpi, with relative mutant abundance being significantly reduced up to 65-fold. As TraDIS analyzed a pool of mixed mutants grown in competition, reduction in mutant survival by a similar degree may not be reproduced in an experiment that involved infection with only a single strain. A decrease in bacterial survival *in vivo* may be associated with a general growth defect. When grown in LB and M9 minimal media, there was no significant difference in growth rate observed for the two mutant strains as compared to the wild type (WT) (**Supplementary Figure S4**), thus confirming that reduced intestinal colonization by Δ*bpsl3313* (at 6 hpi) and *bpsl2988*::Tn*5* (at both 6 and 24 hpi) was not due to defects in bacterial growth or enhanced sensitivity to limited nutrient availability.

**Figure 7:**
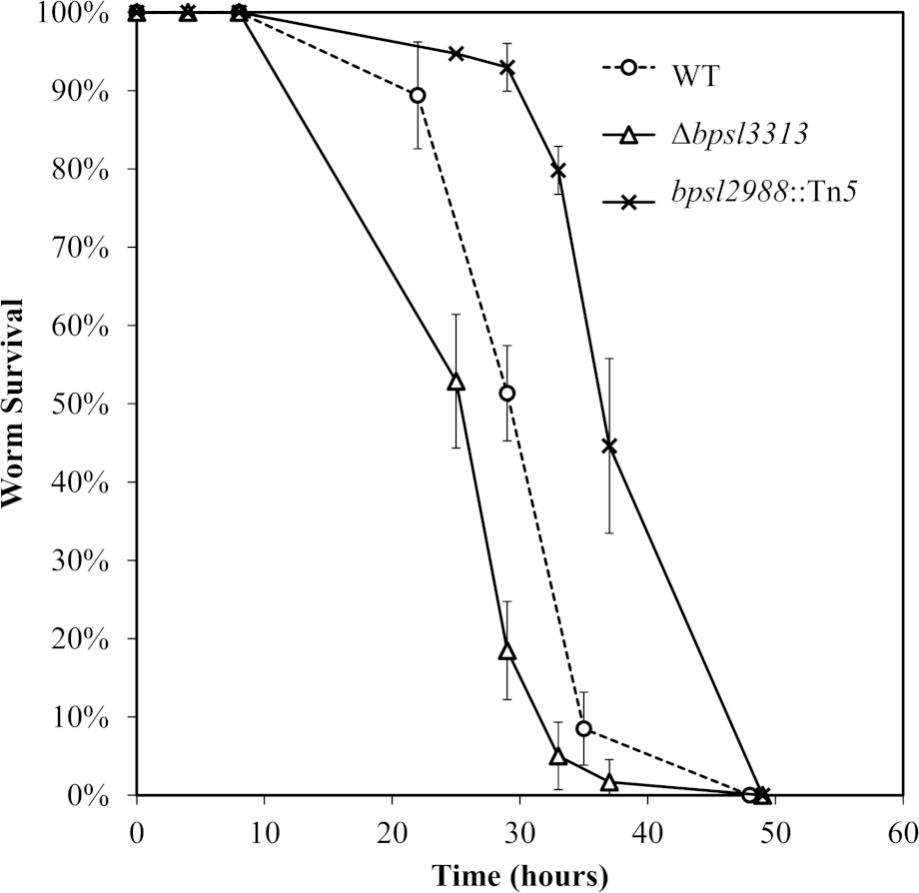
Survival of *C elegans* infected with *B. pseudomallei* wild type (WT) and mutants. Graph shows the mean ± SD from a representative of three independent assays.

The ability of Δ*bpsl3313* and *bpsl2988*::Tn*5* mutants to kill the nematode was evaluated in a standard slow-killing assay. The mean time to death (TD_mean;_ the time required to kill 50% of the worm population) of infected young worms was determined. *bpsl2988*::Tn*5* was strongly attenuated (**Figure 7**) with a TD_mean_ )of 41.07 ± 0.72 hours compared to the wild type (TD_mean_ 32.47 ± 0.62 hours) (Log-rank (Mantel-Cox) test, *p*<0.0001). Surprisingly, the Δ*bpsl3313* mutant was more virulent with a significantly shorter TD_mean_ of 28.26 ± 0.39 hours compared to the wild type (Log-rank (Mantel-Cox) test, p<0.0001).

### 3.7 Mice survival and organ colonization

**Figure 8:**
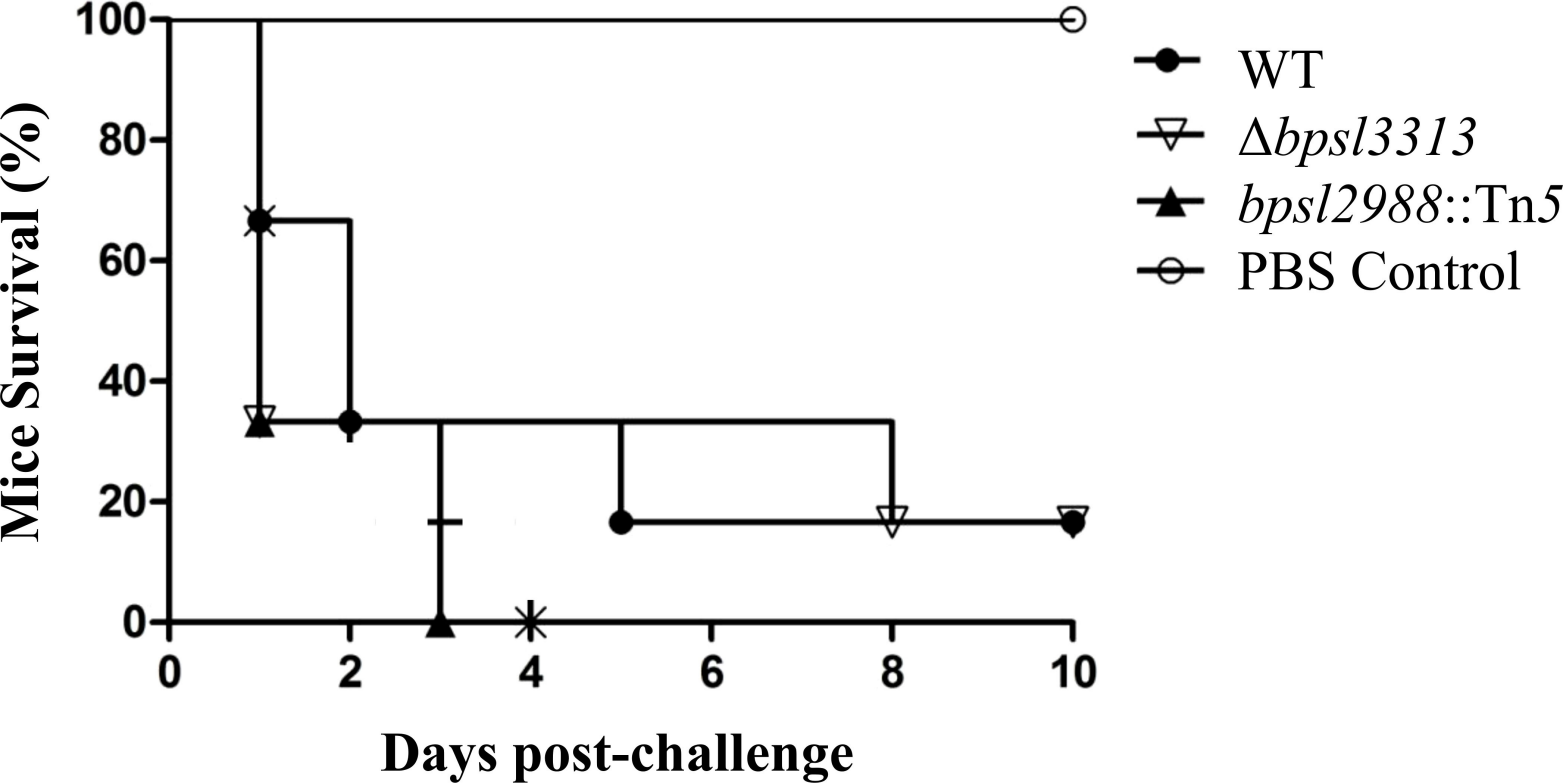
Survival of BALB/c mice infected with *B. pseudomallei* wild type (WT) and mutants. For each strain, mice (n=6) were challenged intraperitoneally and survival was monitored. No significant difference was observed between WT and mutants (Log-rank (Mantel-Cox) test, *p*>0.05).

**Figure 9:**
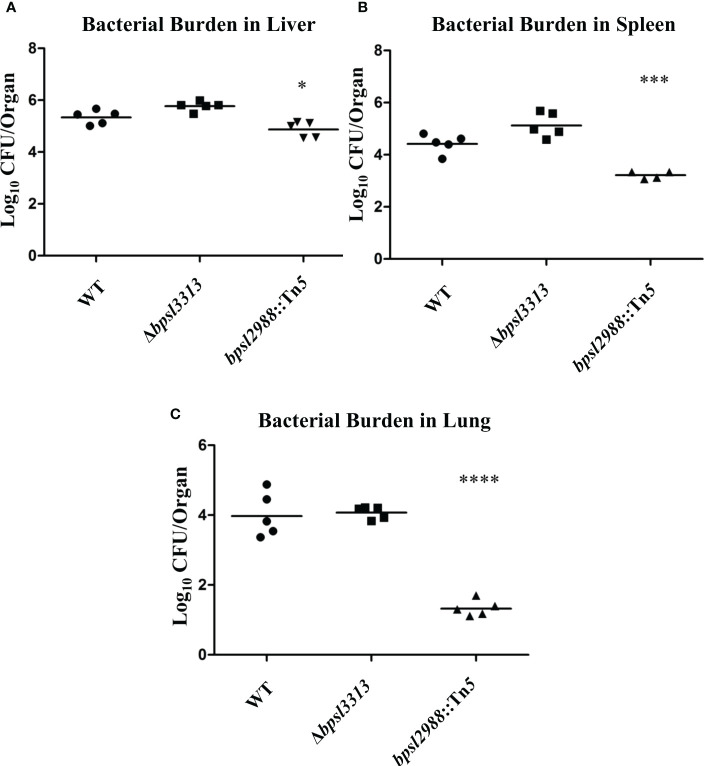
Accumulation of *B. pseudomallei* wild type (WT) and mutants in the **(A)** livers, **(B)** spleens and **(C)** lungs of infected mice at 24 hours post-challenge. Each marker corresponds to the average bacterial CFU (log_10_) extracted from one infected mouse with horizontal lines signifying the geometric mean. The asterisks represent *p* values of the WT counts versus the mutants (*t*-test, *p<0.05; ***p<0.001; ****p<0.0001).

As different model hosts respond to *B. pseudomallei* infection differently (Lee et al., 2011), the well-established BALB/c mouse model for melioidosis was challenged with wild type *B. pseudomallei*, Δ*bpsl3313* and *bpsl2988*::Tn*5* mutants. Mice survival was monitored over time. Unlike the results obtained in a *C. elegans* model, where Δ*bpsl3313* and *bpsl2988*::Tn*5* exhibited different levels of virulence, these two mutants did not show any altered phenotype in mice. More than 60% of the mice infected with either mutants or wild type *B. pseudomallei* succumbed to the disease on day 2 (**Figure 8**). This conflicting response between mice and *C. elegans* may be attributed to distinct mechanisms of host-pathogen interaction between *B. pseudomallei* and both animal models and different hosts’ immune response. Nonetheless, while retaining full virulence, *bpsl2988*::Tn*5* could not colonize the organs of infected mice to the same extent as wild type *B. pseudomallei*. Bacterial burden in the organs of *bpsl2988*::Tn*5*-infected mice were significantly reduced, with the most pronounced colonization defect observed in the lung (*t*-test, *p*<0.0001) (**Figure 9**). Conversely, Δ*bpsl3313* did not show any reduced colonization in the infected mice organs as compared to the wild type. Again, this highlights the important role of BPSL2988 in *B. pseudomallei* survival *in vivo*.

## 4 Discussion

Bacteria are able to detect and respond to environmental cues and this ability has allowed bacteria to adapt to survive in different niches. *B. pseudomallei* is a highly versatile pathogen in that it can adapt and survive in soil, water, infected host and even in the absence of nutrients. The bacterium has a large genome spread over two chromosomes containing genes that encode for proteins with important roles for survival in diverse environments as well as in the infected host. In this study, TraDIS-derived data provided the conditionally essential gene set required for *B. pseudomallei* growth under minimally supplemented growth conditions. When nutrient availability is low, *B. pseudomallei* relied on *de novo* biosynthesis pathways for primary metabolites such as amino acids, nucleotides, and many cofactors like NADP and biotin, all of which are important for the growth and development of the organism. The conditionally essential genes crucial for bacteria to grow under *in vitro* conditions provides a starting point for further studies to delineate promising functions which can be exploited as drug targets. We further identified genes that benefit *B. pseudomallei* survival in the *C. elegans* model infection host. Among these genes are those involved in LPS O-antigen biosynthesis, *de novo* amino acids and nucleic acids biosynthesis, responses against various forms of stress (e.g., oxidative stress) and defense against host immune effectors such as RND efflux pumps.

The essential genomes of different strains of *B. pseudomallei* (R15 and K96243) overlap but are distinct and this feature should enable development of strain-independent drugs. A comparison of the essential genes identified in three Burkholderia species (*B. pseudomallei*, *B. cenocepacia* and *B. thailandensis*) has revealed a core set of genes enriched for DNA transcription, replication, protein translation as well as cell wall biogenesis. Whilst species-specific essential proteins are appealing drug targets, the similarities and differences between essential protein functions may also contribute to the understanding of the evolutionary relationships between Burkholderia species. In *B. pseudomallei* and *B. thailandensis*, genes within the *fab* gene cluster (*acpP*, *fabD*, *fabF*, *fabG*, and *fabH*) are essential for viability. The *fab* gene cluster is involved in fatty acid biosynthesis and the encoded proteins are important in the fatty acid elongation processes of condensation, reduction and dehydration reactions (Kutchma et al., 1999). AcpP is essential for *E. coli* growth (De Lay and Cronan, 2006) and deletion of *fabD* and *fabG* in *E. coli* has been shown to be lethal (Verwoert et al., 1994; Zhang and Cronan, 1998).

Given that the essentiality of a gene is environment-dependent, *B. pseudomallei* rely on *de novo* biosynthesis of essential metabolites (e.g. amino acids, nucleotides and cofactors) to sustain their survival in nutrient-poor environments. The condition-specific essential genes identified in this study provide a general overview on how *B. pseudomallei* is able to adapt to nutrient limitation and forms a strong foundation for future studies on how nutritional cues induce or suppress *B. pseudomallei* pathogenicity during an infection of the host. When the study was extended to an *in vivo* setting, the identification of genetic determinants associated with *B. pseudomallei in vivo* fitness provided an overview of the adaptive strategies exploited by the pathogen in response to adverse *in vivo* environments presented by the infected host. A majority of the genes in the capsule polysaccharide (CPS) (*wcb*) operon were deemed essential for *B. pseudomallei* survival in the *C. elegans* intestinal lumen. These CPS genes, when disrupted, negatively impacted the fitness of the mutant in the worm intestine with different fold change in the relative mutant abundance observed across the CPS gene cluster (**Supplementary Data Table 4**). Gutierrez et al. (2015) also demonstrated that insertions in the CPS locus reduced the relative abundance of mutants in the lungs of mice infected with a *B. pseudomallei* transposon library. Mutants lacking the *wcb* operon were attenuated in a murine respiratory model, yet, able to colonize the lung, liver, and spleen at similar levels comparable to wild type (Warawa et al., 2009). It is likely that during infection of *C. elegans*, the capsule plays an essential role in protecting intestinal bacteria against the host immune system. Three genes in the O-antigen biosynthesis operon (*rmlB*, *rmlD* and *wbiC*) were deemed essential for *B. pseudomallei in vivo* survival by the TraDIS screen. *rmlB* (*bpsl2686*) and *rmlD* (*bpsl2683*) are required for the conversion of dTTP and D-glucose-1-phosphate into dTDP-L-rhamnose, which is then presumably epimerized into talose for incorporation into the LPS O-antigen; whilst *wbiC* (*bpsl2678*) encodes a glycosyltransferase that may catalyze the transfer of sugar residues from nucleotide sugar precursors to und-P-P-Glc*p*NAc, the membrane-bound acceptor (DeShazer et al., 1998). It is evident that LPS O-antigen, stress response regulators such as alternative sigma factors RpoS and RpoE and RND efflux pump are universally required for *B. pseudomallei* survival and adaptation in the *C. elegans* intestinal lumen.

The TraDIS screen also identified a nucleoid-associated protein histone-like nucleoid-structuring (H-NS) protein encoded by *bpsl3313.* At 6 hours and 24 hours post-infection, insertions in *bpsl3313* resulted in reduction of relative mutant abundance by 65-fold. H-NS has been proposed as a “universal repressor” where its family members are involved in regulating bacterial pathogenicity in many different bacterial species (Dillon and Dorman, 2010). The role of H-NS in the negative modulation of virulence-associated genes has been demonstrated in many pathogens such as uropathogenic *E. coli* (Müller et al., 2006), *V. cholerae* (Nye et al., 2000; Wang et al., 2015) and *S. enterica* Typhimurium (Brunet et al., 2015). In *V. cholerae*, deletion of *hns* resulted in increased expression of the virulence-associated genes encoding cholera toxin, toxin-coregulated pilus, virulence gene regulatory protein ToxT (Nye et al., 2000) as well as exopolysaccharide (vps) biosynthesis proteins (Wang et al., 2012). In *S. enterica* Typhimurium, H-NS is a repressor of a T6SS gene cluster located in the SPI-6 pathogenicity island (Brunet et al., 2015). Hence, it is likely that the H-NS protein encoded by *bpsl3313* is likewise involved in the negative regulation of *B. pseudomallei* virulence genes. Further investigations into the BPSL3313 protein should facilitate an understanding of the role of this protein in *B. pseudomallei* pathogenicity.

*bpsl2988* encodes a sugar kinase that is homologous to adenosine kinase (ADK) of the PfkB family of carbohydrate kinases, which has also been reported in *M. tuberculosis* (Meena et al., 2010) and *Xanthomonas campestris* (Lu et al., 2009). ADK, a purine salvage enzyme, catalyses the phosphorylation of adenosine to adenosine monophosphate (AMP) and has been well-characterized in many eukaryotes. As prokaryotes primarily synthesize purines *de novo*, ADK is thought to be rare in bacteria. A seminal study by Park et al. (2009) showed that *M. tuberculosis* ADK behaves very differently from mammalian ADKs in various respects and it is unable to metabolize different adenosine analogues in cellular systems (Park et al., 2009). In *X. campestris*, ADK is important for cell motility, production of extracellular polysaccharide (EPS), and for pathogenicity. *adk* mutants exhibited significant reduction in bacterial growth and virulence in infected host plants (Lu et al., 2009). Further investigation is needed to elucidate the function of the potential adenosine kinase encoded by *bpsl2988*.

This research has provided novel insights into the molecular basis of mechanisms employed by *B. pseudomallei* to grow under different *in vitro* conditions and to survive or persist within different hosts. A number of genes that were identified as essential for *B. pseudomallei* infection and survival in the model host were classified as hypothetical or of unknown function. While this was not surprising as the *B. pseudomallei* genome has not been fully annotated, these genes may code for Burkholderia spp. or *B. pseudomallei* specific proteins which would serve as attractive targets for drugs or vaccine design. These genes may also encode novel virulence factors that contribute to *B. pseudomallei* pathogenicity. Together, these findings warrant further investigation into the precise identity of all the *B. pseudomallei* essential proteins and the mechanisms in which these candidate proteins are involved.

## Data availability statement

The datasets presented in this study can be found in online repositories. The names of the repository/repositories and accession number(s) can be found in the article/**Supplementary Material**.

## Ethics statement

The animal study was reviewed and approved by Universiti Kebangsaan Malaysia Animal Ethics Commitee (UKMAEC), Universiti Kebangsaan Malaysia.

## Author contributions

SN, AP, MA and Y-CW conceived the project and designed the experiments. Y-CW performed the experiments. RN and C-CH performed part of the bioinformatics analysis. Y-CW, MA, AP and SN analyzed the data and wrote the paper. All authors contributed to the article and approved the submitted version.

## Supplementary material

Supplementary Figure 1 Schematic diagram shows the passages of input pool in LB and M9 minimal media (*in vitro*) and *C. elegans* infection model (*in vivo*).

Supplementary Figure 2 Scatter plot showing the correlation between two *B. pseudomallei* replicate libraries. The number of unique insertions (log10) in each gene from Input A was plotted against Input B. A Spearman’s rho correlation coefficient of 0.9855 indicates low variation between replicates.

Supplementary Figure 3 A representative plot presentation the frequency distribution of the gene insertion index. A clear bimodal distribution is displayed where the leftmost peak represents genes in which a transposon insertion would be lethal to the bacteria, while the rightmost peak represents genes in which transposons were able to insert without causing lethality. Bars represent increments of 0.001. Gamma distributions used to estimate likelihood ratios and *p* values are shown with grey lines; the essentiality cut-off is indicated with red lines.

Supplementary Figure 4
*In vitro* growth of *B. pseudomallei* wild type (WT) and mutants Δ*bpsl3313* and *bpsl2988*::Tn*5* in **(A)** LB and **(B)** M9 minimal medium.
