# Supplementary material for: Genome-wide transposon mutagenesis analysis of Burkholderia pseudomallei reveals essential genes for in vitro and in vivo survival
Source: Front Cell Infect Microbiol. 2022 Dec 23;12:1062682. doi: 10.3389/fcimb.2022.1062682 (PMC9816413; doi:10.3389/fcimb.2022.1062682)
Supplement: Supplementary file 1 [file Table_1.docx]

Supplementary Table 1

Table S1 Sequences of oligo/primers used in TraDIS experiment and construction of *bpsl3313* deletion mutant

| **Oligo/primer** | **Sequence (5’ to 3’)** | | **Description** |
| --- | --- | --- | --- |
| **TraDIS** |  | |  |
| Tnp_Fp | TTGGCATGGATTGTAGGCG | | Validation of transposon insertion |
| Tnp_Rp | CAACCCTGAAGCTTGCATGCC | |  |
| MP_ Ad_a | GTGACTGGAGTTCAGACGTGTGCTCTTCCGATC*T | | Adapter ^a^ |
| MP_ Ad_b | p-GATCGGAAGAGCGTCGTGTAGGGAAAGAGTG-amino | |  |
| Tra_Fp | AATGATACGGCGACCACCGAGATCTACACCTGATCTAGAGTCGACCTGCAGGCATGCAAGCTTCAG | | Amplification of TraDIS library; Forward and reverse primer^b^ |
| Tra_Mp_Rp | CAAGCAGAAGACGGCATACGAGATNNNNNNNGTGACTGGAGTTCAGACGTGT | |  |
| qPCR_P5 | AATGATACGGCGACCACCGA | | TraDIS library quantification by qPCR |
| qPCR_P7 | CAAGCAGAAGACGGCATACGA | |  |
| Tra_SeqP | AGGCATGCAAGCTTCAGGGTTGAGATGTGTA | | Sequencing primer |
| Tra_IndP | GATCGGAAGAGCACACGTCTGAACTCCAGTCAC | | Index primer |
| **Deletion Mutant Construction** ^c^ | | |  |
| *bpsl3313*_US  forward primer | | GTGAAAATTGAAAACGGATGGCG | Amplification of upstream (US) of *bpsl3313* gene |
| *bpsl3313*_US  reverse primer | | ATCATGATCCGCCTGGCATT |  |
| *bpsl3313*_DS  forward primer | | AATGCCAGGCGGATCATGATGCATTGGATCGTCGGCAAAA | Amplification of downstream (DS) of *bpsl3313* gene |
| *bpsl3313*_DS  reverse primer | | ATTCGAGCAACGTTTCAGCTC |  |
| *pEXKm5 OriT forward primer* | | TCCGCTGCATAACCCTGCTTC | Validation of pEXKm5 |
| *pEXKm5 OriT*  *reverse primer* | | CAGCCTCGCAGAGCAGGATTC |  |

^a^Both oligonucleotides were modified from the Illumina adapter. The asterisk indicates a phosphorodioate bond, p represents 5’ phosphate modifier and amino represents 3’ amino modifier. Underlined is the 12-bp complementary region.

^b^N signifies 7 bp barcodes as described by Meyer and Kircher (2010).

^c^Deletion mutant constructed as described by Wong et al. (2018).
