# Supplementary figures and images for: Genome-wide transposon mutagenesis analysis of Burkholderia pseudomallei reveals essential genes for in vitro and in vivo survival
Source: Front Cell Infect Microbiol. 2022 Dec 23;12:1062682. doi: 10.3389/fcimb.2022.1062682 (PMC9816413; doi:10.3389/fcimb.2022.1062682)

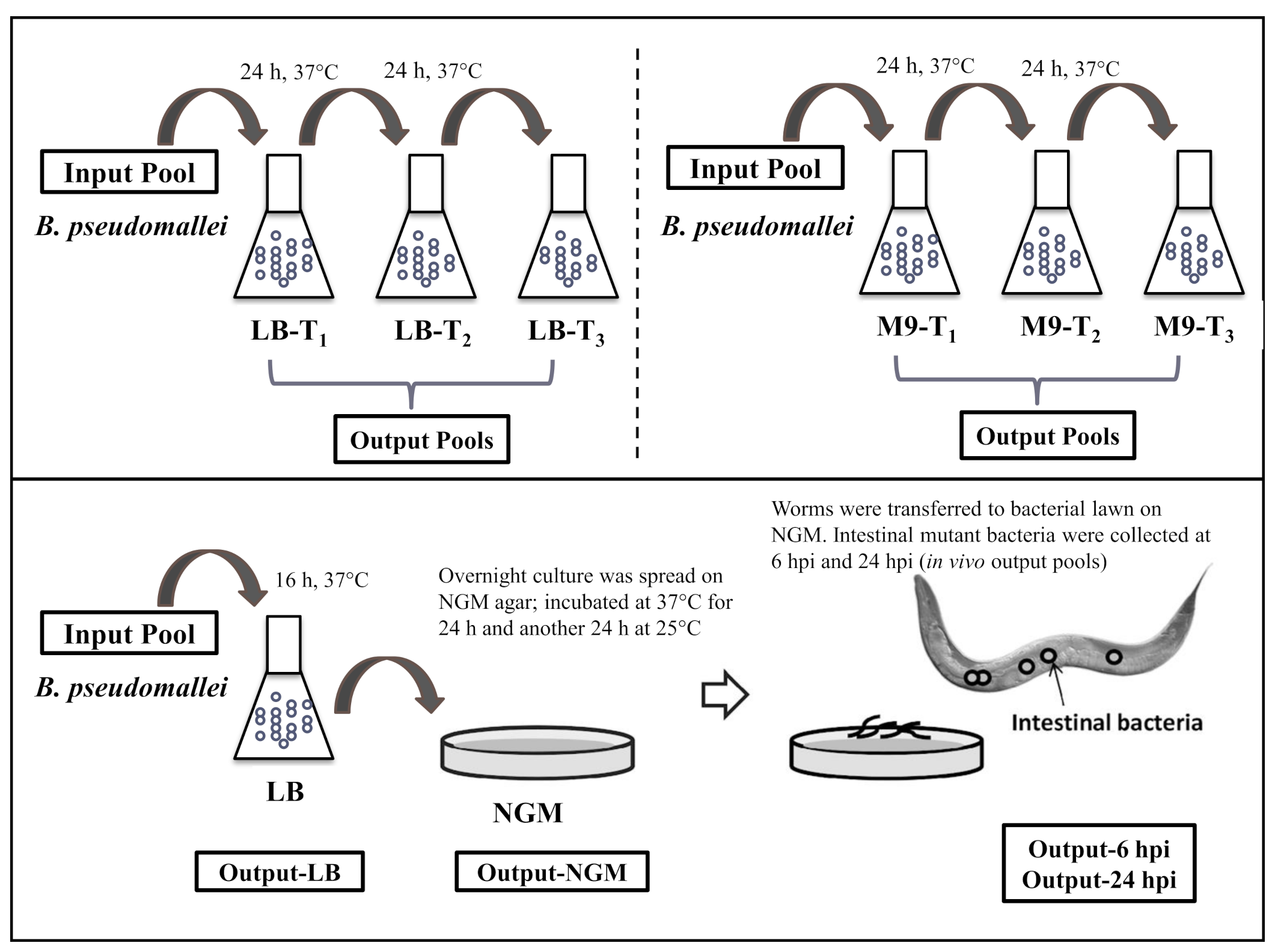

Supplement: Supplementary Figure 1 — Schematic diagram shows the passages of input pool in LB and M9 minimal media (in vitro) and C. elegans infection model (in vivo). [file Image_1.tif]

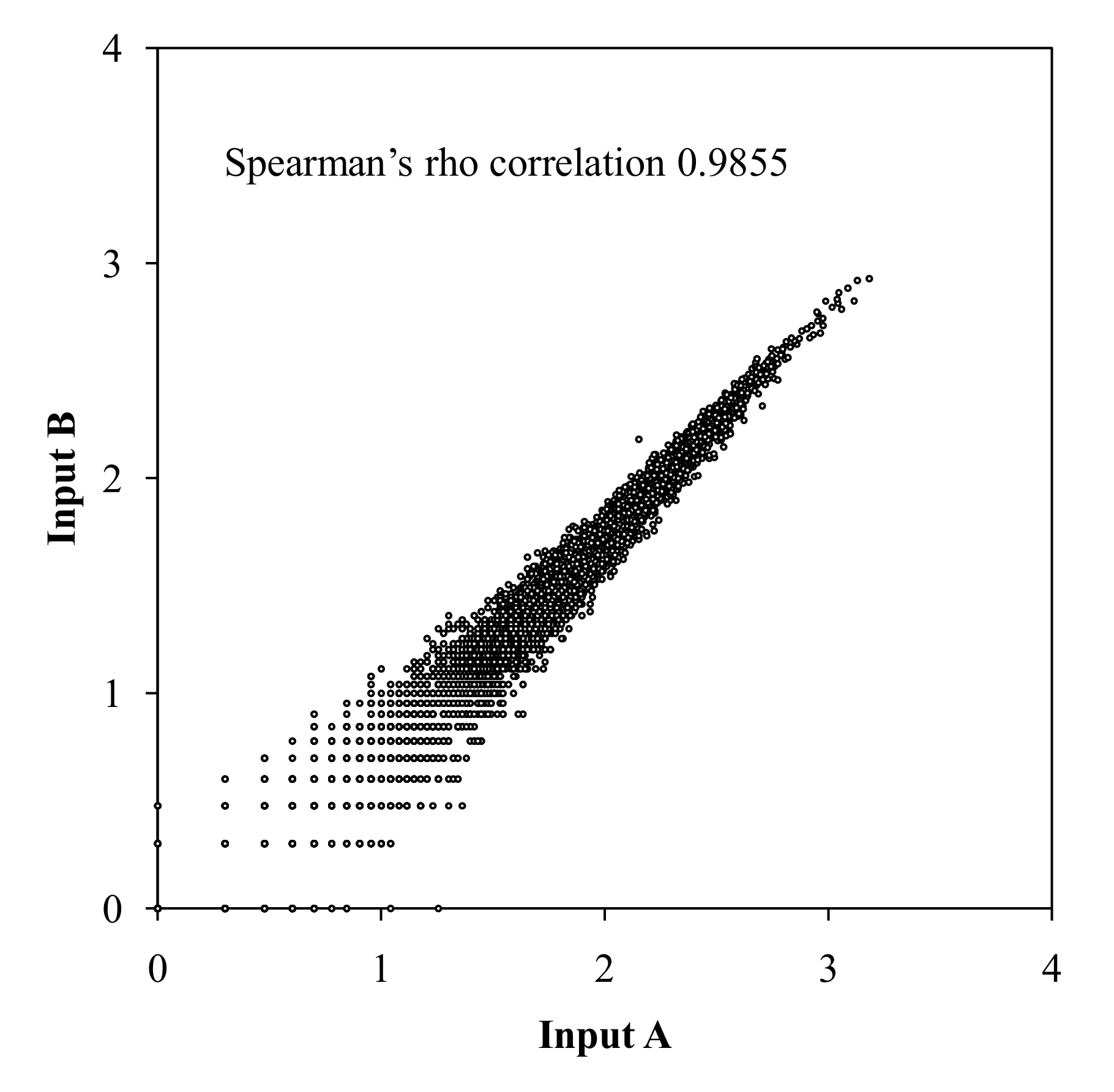

Supplement: Supplementary Figure 2 — Scatter plot showing the correlation between two B. pseudomallei replicate libraries. The number of unique insertions (log10) in each gene from Input A was plotted against Input B. A Spearman’s rho correlation coefficient of 0.9855 indicates low variation between replicates. [file Image_2.tif]

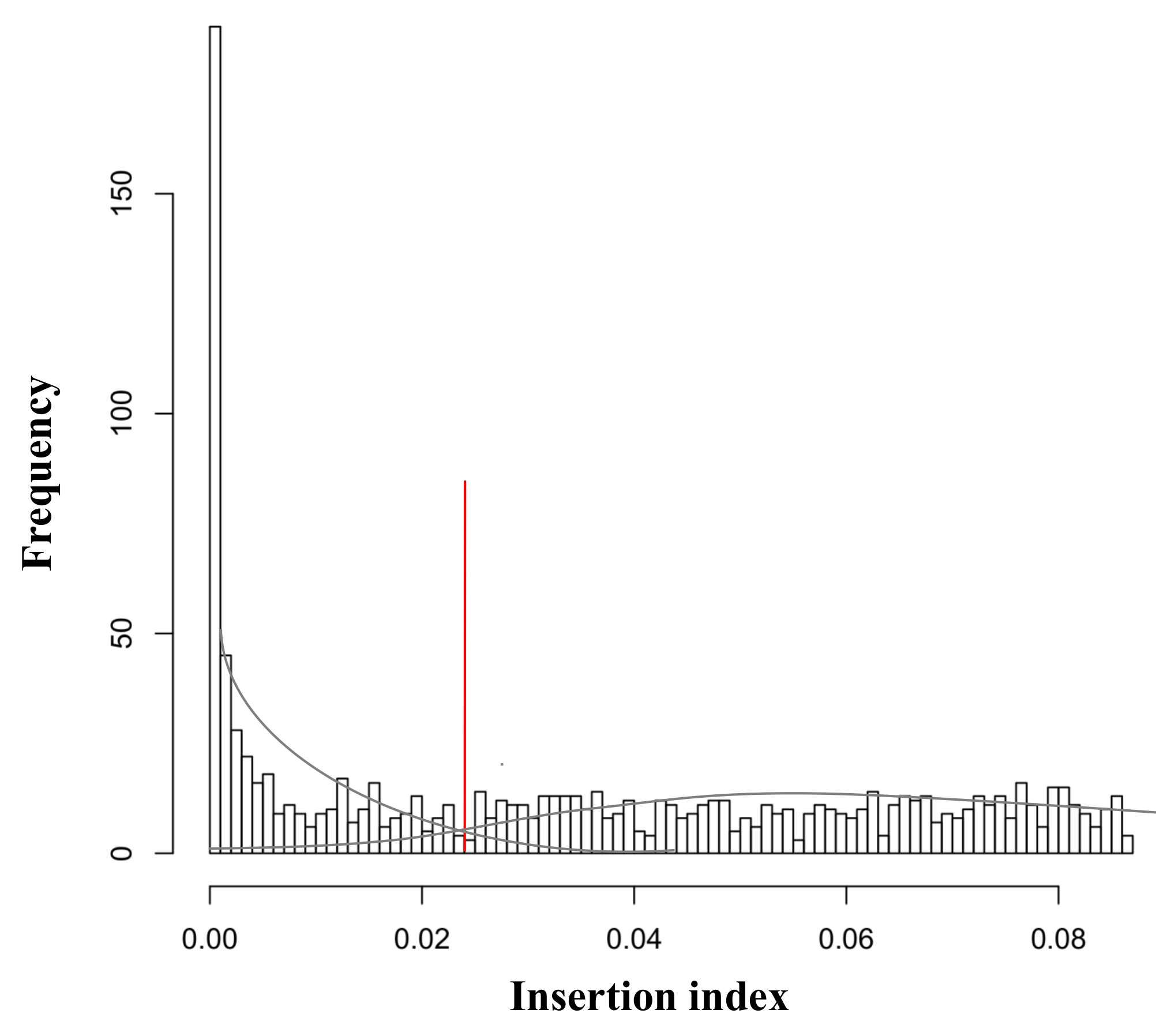

Supplement: Supplementary Figure 3 — A representative plot presentation the frequency distribution of the gene insertion index. A clear bimodal distribution is displayed where the leftmost peak represents genes in which a transposon insertion would be lethal to the bacteria, while the rightmost peak represents genes in which transposons were able to insert without causing lethality. Bars represent increments of 0.001. Gamma distributions used to estimate likelihood ratios and p values are shown with grey lines; the essentiality cut-off is indicated with red lines. [file Image_3.tif]

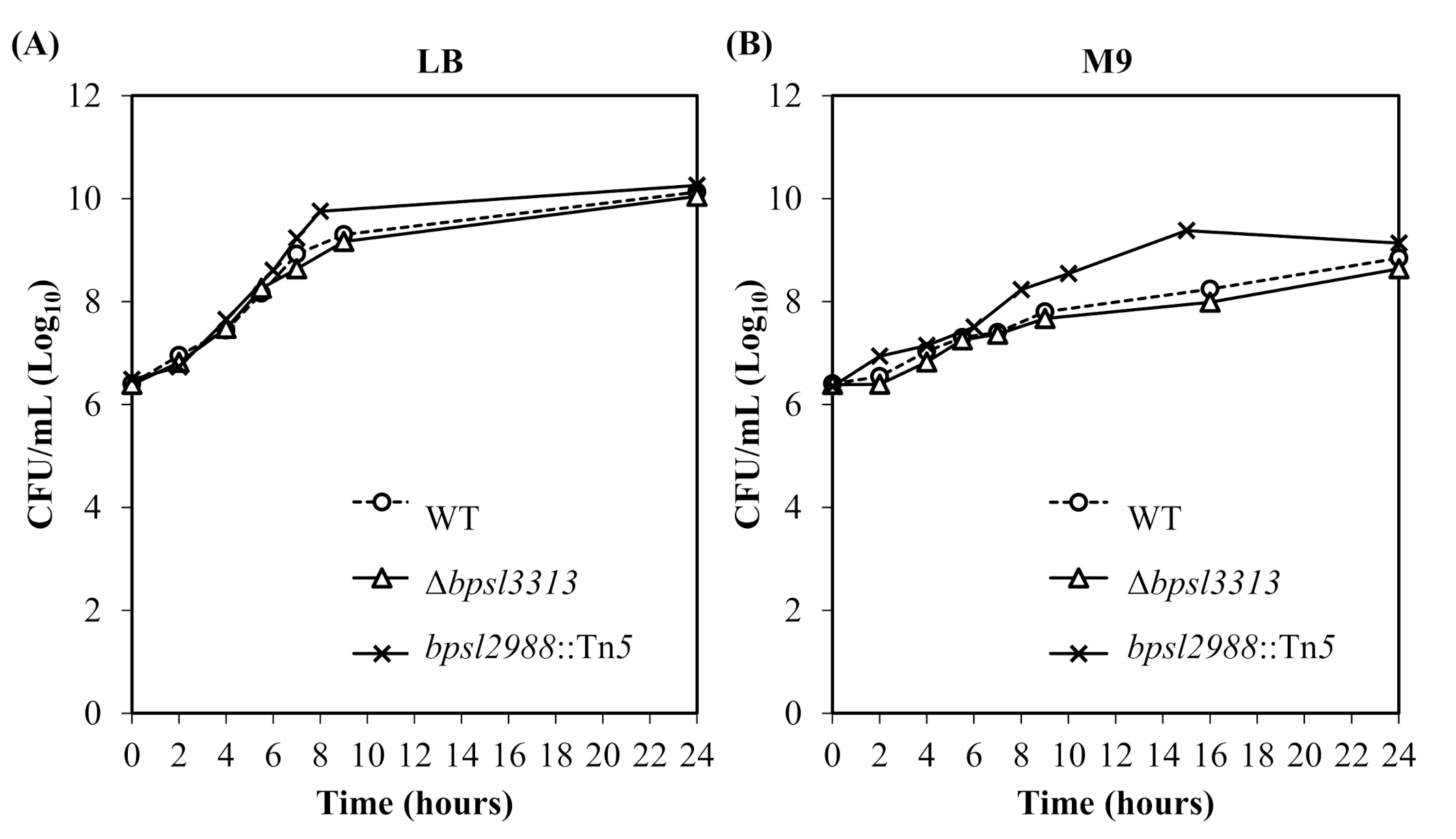

Supplement: Supplementary Figure 4 — In vitro growth of B. pseudomallei wild type (WT) and mutants Δbpsl3313 and bpsl2988::Tn5 in (A) LB and (B) M9 minimal medium. [file Image_4.tif]
